# Supplementary material for: PARP2 promotes Break Induced Replication-mediated telomere fragility in response to replication stress
Source: Nat Commun. 2024 Apr 2;15:2857. doi: 10.1038/s41467-024-47222-7 (PMC10987537; doi:10.1038/s41467-024-47222-7)
Supplement: Supplementary file 1 — Supplementary Information [file 41467_2024_47222_MOESM1_ESM.pdf]

Supplementary figure 1

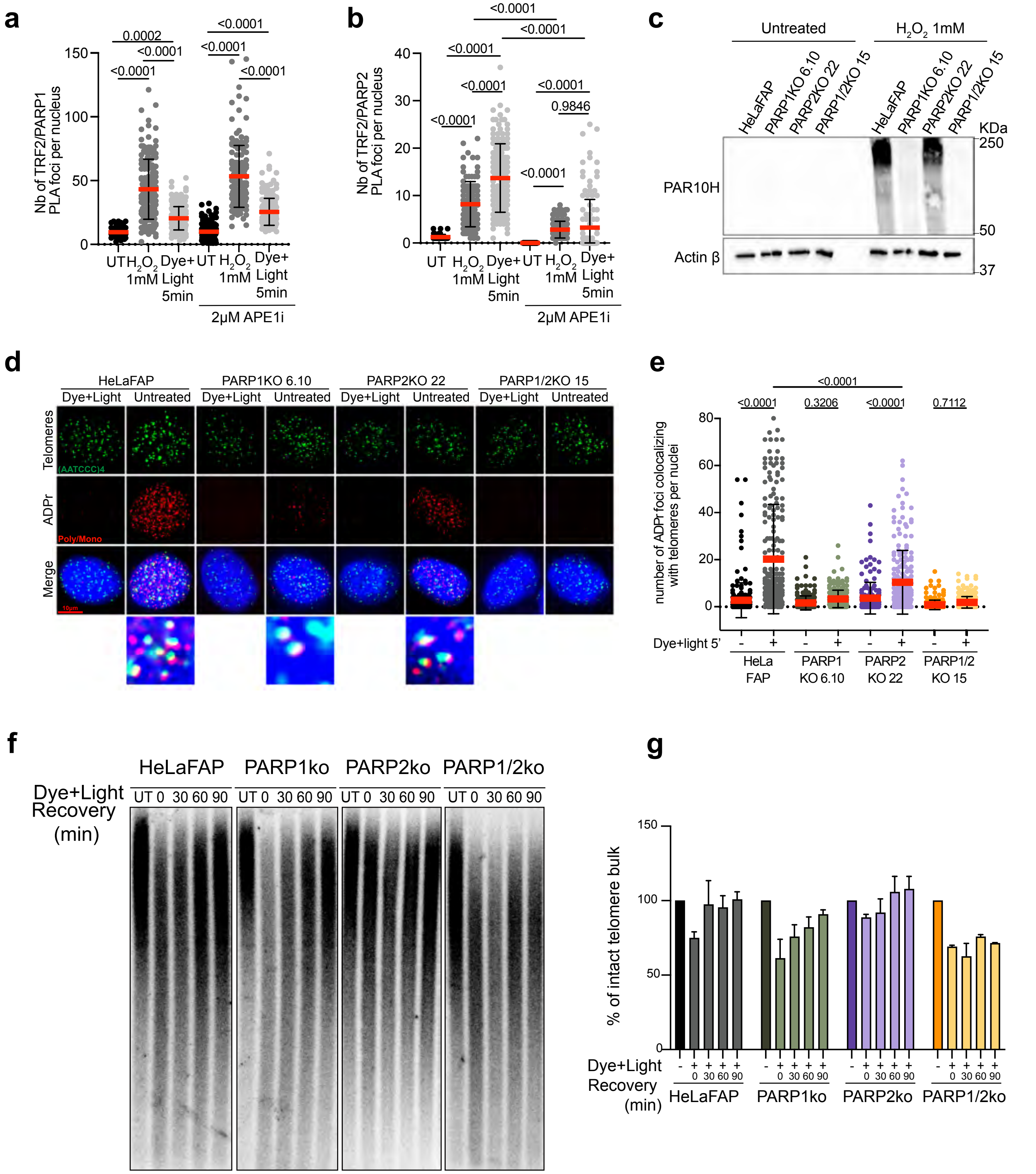

Supplementary Figure 1: BOTH PARP1 AND PARP2 CONTRIBUTE TO OXIDATIVE LESION REPAIR AT TELOMERES.

(a-b) Quantification of the number of PLA TRF2:PARP2 (a) and TRF2:PARP1 (b) foci (red) per nucleus detected in HeLaFAP cells upon H_2O_2 or after acute dye and light treatment. Cells were pre-treated with an APE1 inhibitor (inhibitor III) prior to dye addition. Each dot on the graph corresponds to a specific analyzed nucleus. At least 150 cells were analyzed per experiment. Red bars represent the mean ± SD from the indicated n number of nuclei analyzed from three independent experiments. P values were obtained using ordinary one-way ANOVA.

(c) Immunoblot showing accumulation of PAR from HeLaFAP, PARP1KO, and PARP2KO cell extracts treated with 1mM H_2O_2 for 15 minutes. Actin was used as a loading control.

(d) Images of PAR (red) and telomeres (green) by anti-ADPr IF and telo-FISH respectively. The last row shows an enlargement of PAR foci colocalizing with telomeres.

(e) Quantification of ADPr foci colocalizing with telomeres in cells treated with dye and light. Each dot on the graph corresponds to a specific analyzed nucleus. At least 300 cells were analyzed. Red bars represent mean ± SD from the indicated n number of nuclei analyzed. P values were obtained using ordinary one-way ANOVA.

(f) Representative image of the detection of 8oxoG-induced SSBs at telomeres after acute treatment with dye and light, by alkaline-denaturing gel and telomere southern blot.

(g) Quantification of the percentage of intact telomeres from (f). Source data are provided as a Source Data file.

Supplementary figure 2

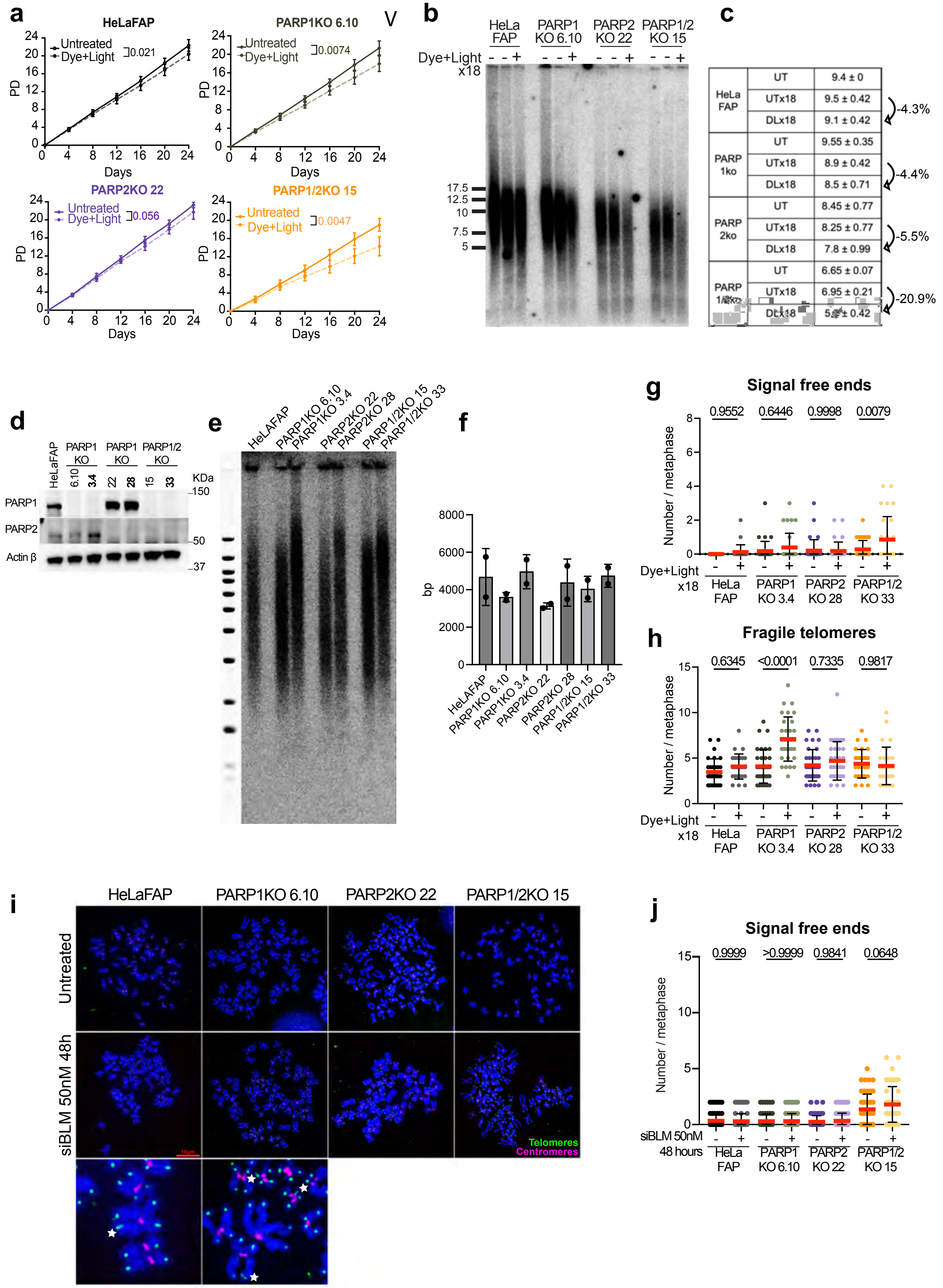

HeLa FAP	UT	9.4 ± 0	
	UTx18	9.5 ± 0.42	
	DLx18	9.1 ± 0.42	-4.3%
PARP 1ko	UT	9.55 ± 0.35	
	UTx18	8.9 ± 0.42	
	DLx18	8.5 ± 0.71	-4.4%
PARP 2ko	UT	8.45 ± 0.77	
	UTx18	8.25 ± 0.77	
	DLx18	7.8 ± 0.99	-5.5%
PARP 1/[illegible]	UT	6.65 ± 0.07	
	UTx18	6.95 ± 0.21	
	DLx18	5[illegible] ± 0.42	-20.9%

Supplementary Figure 2: PARP2 DEPLETION PREVENTS REPLICATION STRESS MEDIATED TELOMERE FRAGILITY

(a) Population doubling (PD) over 24 days of untreated and chronic dye and light-treated cells. Means ± SD from 5 to 8 independent experiments. P values were obtained using Welch's two-sample t test.

(b) Southern blot of telomere restriction fragment length analysis from the indicated cell lines harvested 24 hours after treatment on day 24 (18 treatments) compared to cells harvested at day 0 or untreated cells on day 24.

(c) Table of TRF analysis of (b), with the percentage of telomere mean differences between treatments.

(d) Immunoblot of PARP1 and PARP2 in parental HeLaFAP cells and the KO clones. Additional clones are highlighted. Actin was used as a loading control.

(e) PFGE and Southern blot of telomere restriction fragments from HeLaFAP and PARP1KO and PARP2KO genomic DNA.

(f) Mean telomere length of HeLaFAP, PARP1KO, and PARP2KO cells from (E). Error bars represent mean ± SD from 2 independent experiments.

(g) Quantification of telomeric signal-free ends 24 hours after the 18th dye and light exposure of PARP1KO 3.4, PARP2KO 28, and PARP1/2KO 33 additional cell lines. Each dot represents a metaphase. Red bars represent mean ± SD from more than 30 metaphases per condition. P values were obtained using ordinary one-way ANOVA

(h) Quantification of fragile telomeres 24 hours after the 18th dye and light exposure of PARP1KO 3.4, PARP2KO 28, and PARP1/2KO 33 additional cell lines. Each dot represents a metaphase. Red bars represent mean ± SD from more than 30 metaphases per condition. P values were obtained using ordinary one-way ANOVA

(i) Representative images of telomere FISH on metaphase chromosomes 48 hours after depletion of BLM with siRNA. Fragile telomeres (white stars) are indicated.

(j) Quantification of telomeric signal-free ends detected by FISH in HelaFAP, PARP1KO, PARP2KO, and PARP1/2KO after knockdown of BLM with siRNA. Each dot represents a metaphase. At least 20 to 30 metaphases were analyzed per experiment. Red bars represent mean ± SD from n metaphases analyzed from two independent experiments. P values were obtained using ordinary one-way ANOVA. Source data are provided as a Source Data file.

Supplementary figure 3

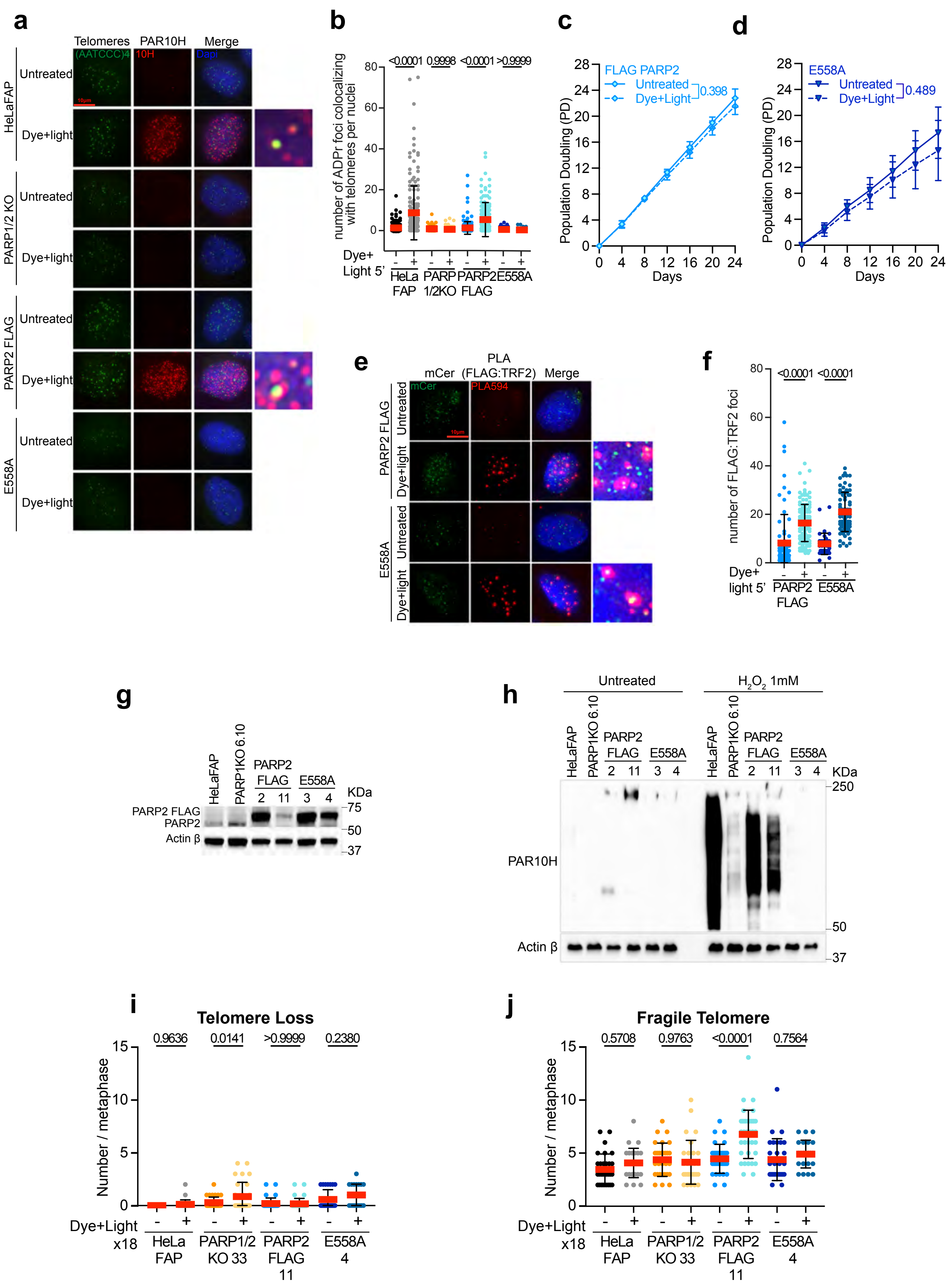

Supplementary Figure 3: TELOMERE FRAGILITY IS MEDIATED BY PARP2 AND ITS CATALYTIC ACTIVITY DURING REPLICATION STRESS

(a) Representative images of PAR (red) and telomeres (green) by anti-PAR IF and telo-FISH respectively in HeLaFAP, PARP1/2KO, PARP2-FLAG, and E558A cells after acute dye and light treatment. The last column shows PAR foci colocalizing with telomeres.

(b) Quantification of PAR foci colocalizing with telomeres. Red bars represent mean ± SD from more than 350 cells per condition.

(c-d) Population doubling (PD) over 24 days of untreated and chronic dye and light-treated PARP2-FLAG and E558A cells. Means ± SD from 3 independent experiments. P values were obtained using Welch's two-sample t test.

(e) Representative images of FLAG:TRF2 PLA foci (in red). PLA signals colocalize with telomeric signals FAP-mCer, shown in green. The last column shows a detailed enlargement of colocalization.

(f) Quantification of the number of PLA FLAG:TRF2 foci per nucleus in FLAG-PARP2, and E558A cells after dye and light treatment. Each dot on the graph corresponds to a specific analyzed nucleus. At least 150 cells per condition were counted. Red bars represent the means ± SD. P values were obtained using ordinary one-way ANOVA.

(g) PARP1/2KO cells were transfected with the pLVX-IRES-puro-PARP2-FLAG plasmid, and its version containing the point mutations, and protein expression level was analyzed in different clones by immunoblotting using an anti-PARP2 antibody. Actin was used as a loading control.

(h) Anti-PAR 10H immunoblot showing accumulation of PAR from HeLaFAP, PARP1KO, PARP2 complemented cell, and the point mutant complemented cells treated with 1mM H_2O_2 for 15 minutes. Actin was used as a loading control.

(i) Quantification of telomeric signal-free ends 24 hours after the last dye and light exposure (N18) of PARP2-FLAG 11 and E558A 4 additional cell lines. Each dot represents a metaphase. Means ± SD from more than 60 metaphases. P values were obtained with ordinary one-way ANOVA. P values were obtained using ordinary one-way ANOVA.

(j) Quantification of fragile telomeres 24 hours after the last dye and light exposure (N18) of PARP2-FLAG 11 and E558A 4 additional cell lines. Each dot represents a metaphase. Means ± SD from more than 60 metaphases. P values were obtained with ordinary one-way ANOVA. P values were obtained using ordinary one-way ANOVA. Source data are provided as a Source Data file.

Supplementary figure 4

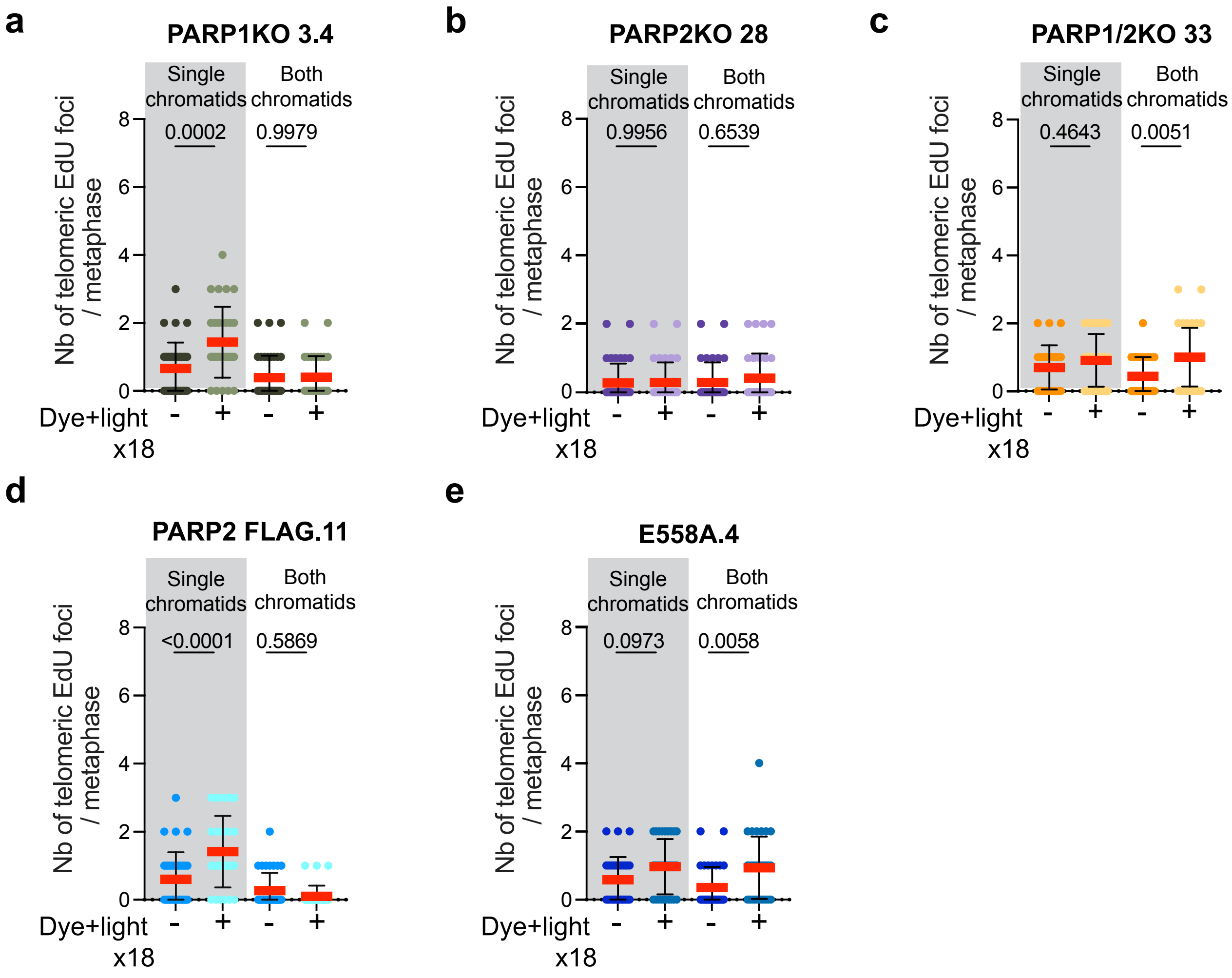

Supplementary Figure 4: PARP2 ORCHESTRATES MITOTIC DNA SYNTHESIS AT TELOMERES UPON REPLICATION STRESS.

(a-e) Quantification of EdU incorporation at telomeres on single or both chromatid ends of metaphase chromosomes from every additional PARP1KO, PARP2KO, PARP1/2KO and complemented cell line after 18 dye and light treatments. Each dot represents a metaphase. At least 30 metaphases were analyzed per experiment. Red bars represent mean ± SD from n metaphases analyzed. P values were obtained using ordinary one-way ANOVA. Source data are provided as a Source Data file.

Supplementary figure 5

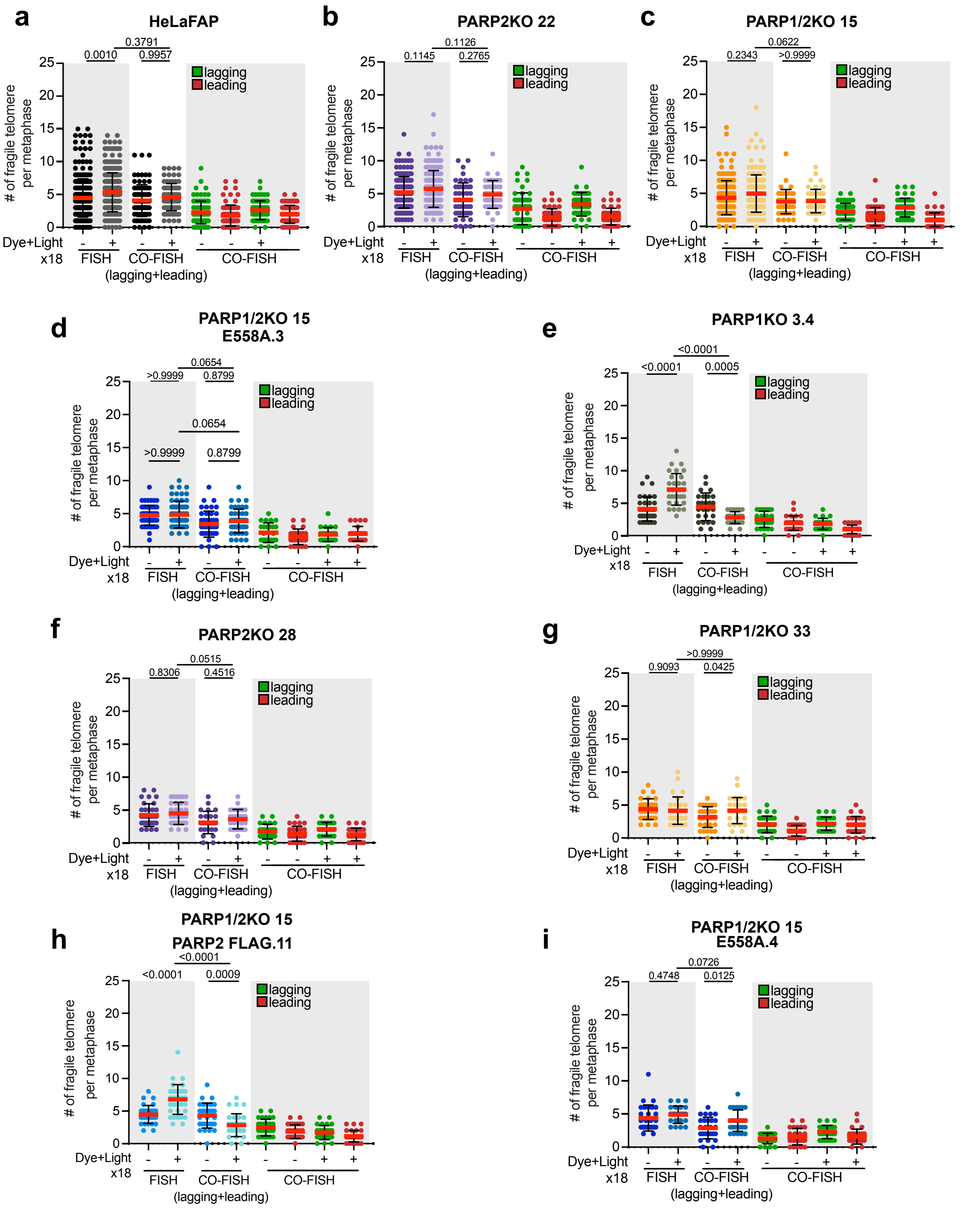

Supplementary Figure 5: PARP2 STIMULATES BIR-MEDIATED DNA END RESECTION

(a-i) Quantification of fragile telomeres in HeLaFAP, PARP1KO (3.4), PARP2KO (22 and 28), PARP1/2KO (15 and 33), PARP2-FLAG (11), and E558A (3 and 4) cells after 18 dye and light treatments detected by FISH compared to fragile telomeres detected by CO-FISH on samples derived from BrdU/BrdC-labeled cells, and quantification of leading- and lagging-end telomeres. Each dot represents a metaphase. At least 30 metaphases were analyzed. Red bars represent mean ± SD from n metaphases analyzed. P values were obtained using ordinary one-way ANOVA. Source data are provided as a Source Data file.

Supplementary figure 6

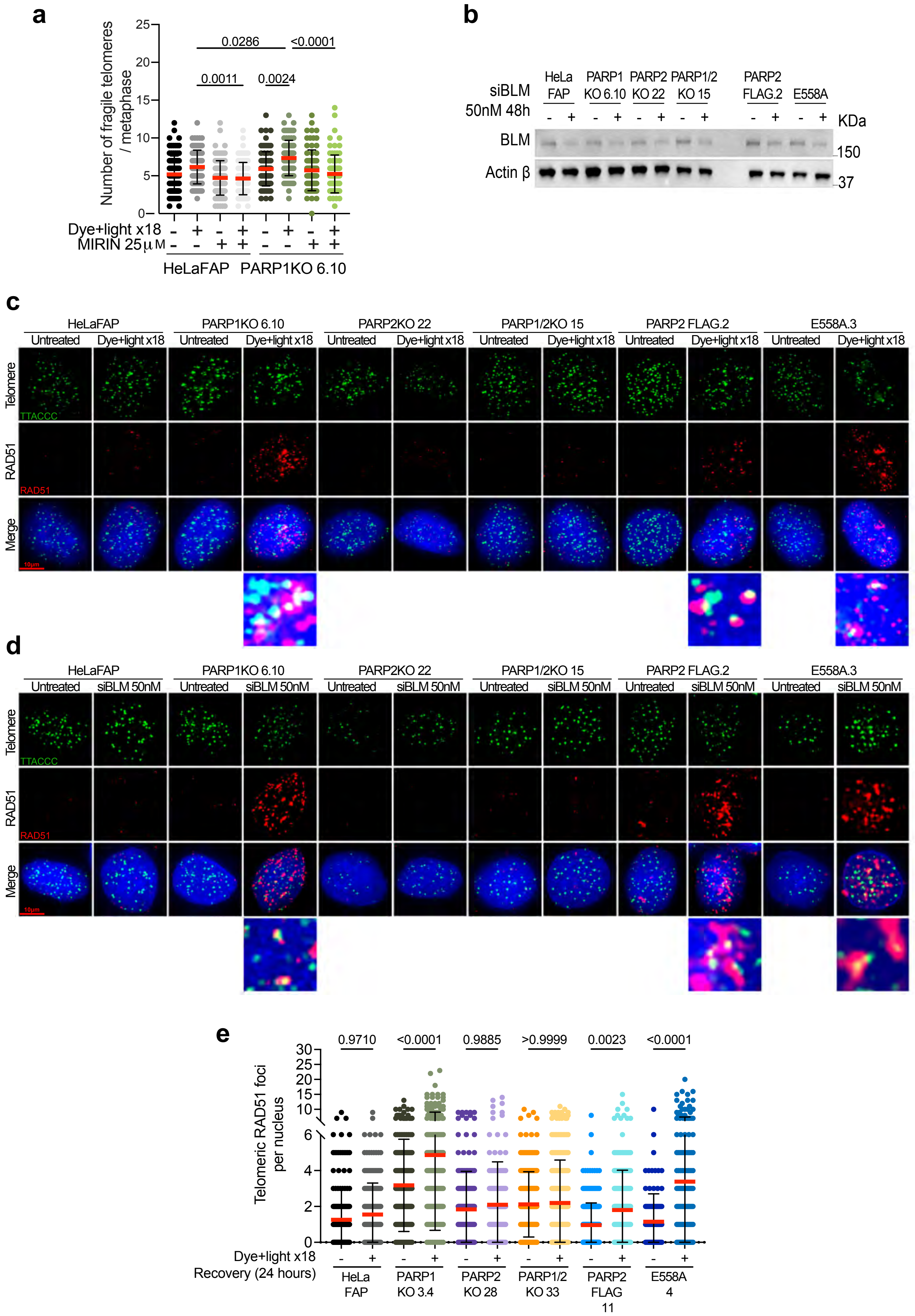

Supplementary Figure 6: PARP2 STIMULATES BIR-MEDIATED DNA END RESECTION

(a) Quantification of fragile telomeres from HeLaFAP and PARP1KO cells treated with the MRN inhibitor, MIRIN, after the last dye and light treatment (N18). Each dot represents a metaphase. At least 20 metaphases were analyzed per experiment. Red bars represent mean ± SD from n metaphases analyzed from three independent experiments. P values were obtained using Two-way ANOVA.

(b) Suppression of BLM expression with BLM siRNA. Western blot of BLM in HelaFAP, PARP1KO, PARP2KO, PARP1/2KO, PARP2-FLAG, and E558A cells treated with 50 nM BLM siRNA for 48 hours. Actin was used as a loading control.

(c) Representative images of RAD51 foci (in red) combined with FISH staining of telomeres (in green) in HeLaFAP, PARP1KO, PARP2ko, PARP1/2ko, FLAG PARP2, and E558A cell lines. Cells were fixed 24 hours after the last dye and light treatment (N18). The last row corresponds to zoomed-in squares showing marked RAD51 foci colocalizing with telomeres.

(d) Representative images of RAD51 foci (in red) combined with FISH staining of telomeres (in green) in HeLaFAP, PARP1KO, PARP2ko, PARP1/2ko, FLAG PARP2, and E558A cell lines. Cells were fixed 48 hours after the knockdown of BLM with siRNA. The last row corresponds to zoomed-in squares showing marked RAD51 foci colocalizing with telomeres.

(e) Quantification of the number of RAD51 foci colocalizing with telomeres per nucleus in additional PARP1 and PARP2KO cell lines. Cells were fixed 24 hours after the last dye and light treatment (N18). Each dot on the graph corresponds to a specific analyzed nucleus. At least 200 cells were analyzed per condition. Red bars represent mean ± SD. Statistical analysis was performed using ordinary one-way ANOVA. Source data are provided as a Source Data file.

Supplementary figure 7

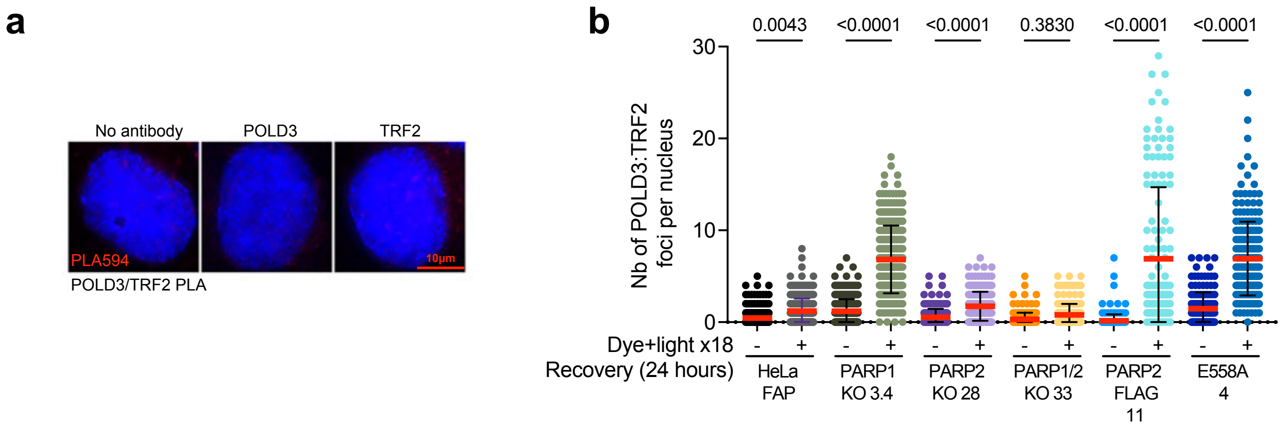

Supplementary Figure 7: PARP2 IS REQUIRED FOR POLD3 RECRUITMENT TO TELOMERES

(a) Representative images of the antibody controls for the POLD3:TRF2 PLA.

(b) Quantification of the number of POLD3:TRF2 PLA foci (red) per nucleus detected in HeLaFAP, PARP1KO 3.4, PARP2KO 28, PARP1/2ko 33, PARP2-FLAG 11 and E558A.4 cells. Cells were fixed 24 hours after the last dye and light treatment (N18). Each dot on the graph corresponds to a specific analyzed nucleus. At least 300 cells were analyzed per condition. Red bars represent mean ± SD. P values were obtained using ordinary one-way ANOVA. Source data are provided as a Source Data file.

Supplementary figure 8

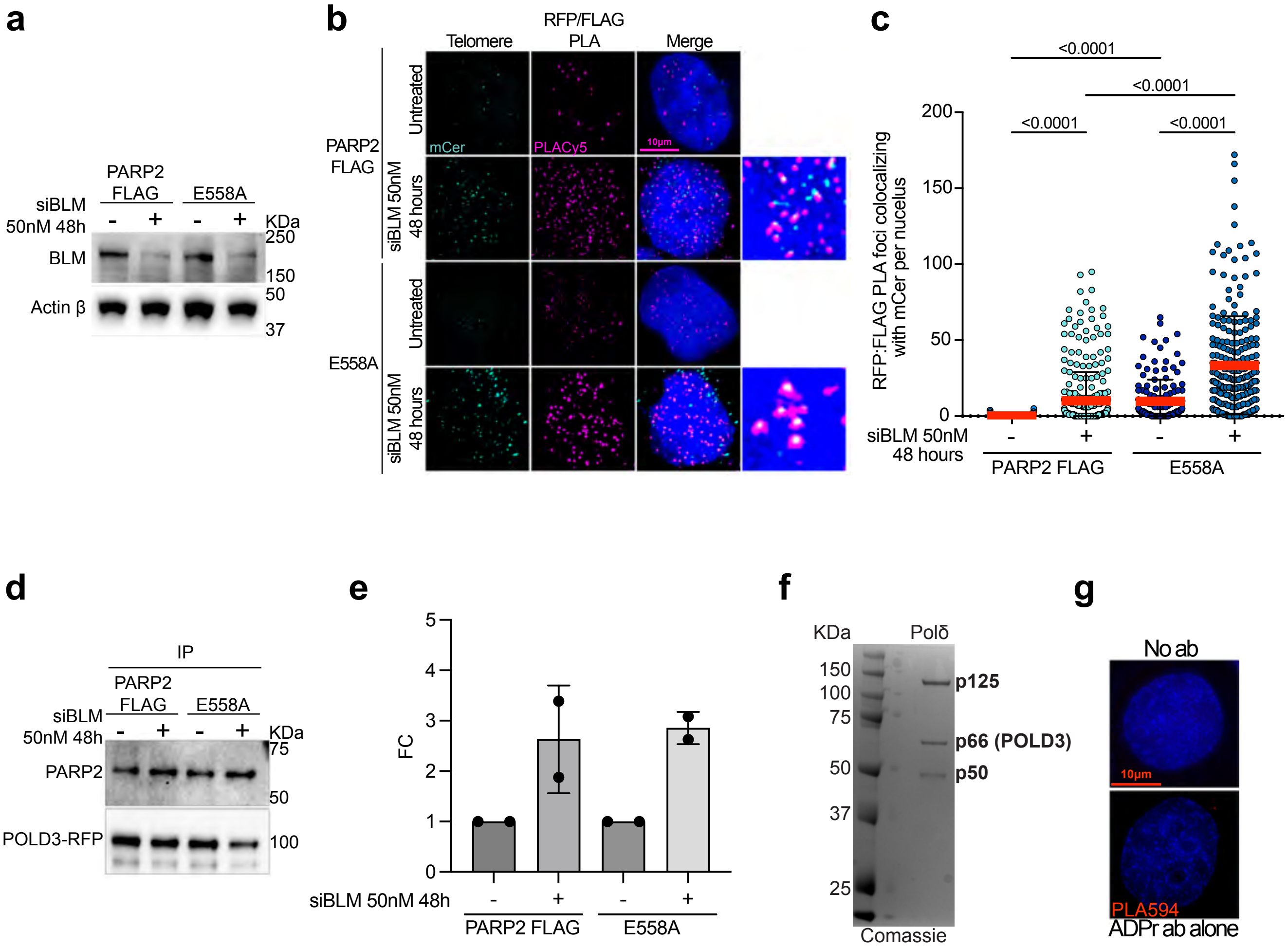

Supplementary Figure 8: POLD3 IS A TARGET OF PARP2

(a) Suppression of BLM expression with BLM siRNA. Western blot of BLM in PARP2-FLAG, and E558A cells treated with 50 nM BLM siRNA for 48 hours. Actin was used as a loading control.

(b) Representative images of RFP:FLAG PLA (pink) and FAP-mCer-TRF1 (cyan) foci in PARP2-FLAG, and E558A cell lines. PLA signals colocalize with telomeric signals FAP-mCer, shown in yellow. The last column shows a detailed enlargement of colocalization.

(c) Quantification of the number of RFP:FLAG PLA foci colocalizing with telomeres per nucleus. Each dot on the graph corresponds to a specific analyzed nucleus. At least 100 to 300 cells were counted per condition. Red bars represent mean ± SD from n nuclei analyzed from two independent experiments. Statistical analysis was performed using ordinary one-way ANOVA.

(d) Co-immunoprecipitation of PARP2 with RPF, in protein extract from cells expressing POLD3-RFP, in response to depletion of BLM using siRNA. Extract preparation and immunoprecipitation were performed with RFP beads and immunoblot was performed using anti-PARP2 and anti-POLD3 antibodies.

(e) Quantification of PARP2 bound to POLD3 in cells depleted for BLM with siRNA from the immunoblot shown in (d). Error bars represent mean ± SD from 2 independent experiments.

(f) Coomassie-stained SDS-PAGE protein gel of purified recombinant human DNA polymerase δ.

(g) Representative images of the antibody controls for the ADPr:POLD3 PLA. Source data are provided as a Source Data file.
