# Supplementary material for: PARP2 promotes Break Induced Replication-mediated telomere fragility in response to replication stress
Source: Nat Commun. 2024 Apr 2;15:2857. doi: 10.1038/s41467-024-47222-7 (PMC10987537; doi:10.1038/s41467-024-47222-7)
Supplement: Supplementary file 3 — Reporting Summary [file 41467_2024_47222_MOESM3_ESM.pdf]

nature portfolio

Corresponding author(s): Elise Fouquerel

Last updated by author(s): 2024-30-01

Reporting Summary

Nature Portfolio wishes to improve the reproducibility of the work that we publish. This form provides structure for consistency and transparency in reporting. For further information on Nature Portfolio policies, see our Editorial Policies and the Editorial Policy Checklist.

Statistics

For all statistical analyses, confirm that the following items are present in the figure legend, table legend, main text, or Methods section.

n/a	Confirmed	
☐	☒	The exact sample size (*n*) for each experimental group/condition, given as a discrete number and unit of measurement
☐	☒	A statement on whether measurements were taken from distinct samples or whether the same sample was measured repeatedly
☐	☒	The statistical test(s) used AND whether they are one- or two-sided<br>*Only common tests should be described solely by name; describe more complex techniques in the Methods section.*
☒	☐	A description of all covariates tested
☒	☐	A description of any assumptions or corrections, such as tests of normality and adjustment for multiple comparisons
☐	☒	A full description of the statistical parameters including central tendency (e.g. means) or other basic estimates (e.g. regression coefficient) AND variation (e.g. standard deviation) or associated estimates of uncertainty (e.g. confidence intervals)
☐	☒	For null hypothesis testing, the test statistic (e.g. *F*, *t*, *r*) with confidence intervals, effect sizes, degrees of freedom and *P* value noted<br>*Give P values as exact values whenever suitable.*
☒	☐	For Bayesian analysis, information on the choice of priors and Markov chain Monte Carlo settings
☒	☐	For hierarchical and complex designs, identification of the appropriate level for tests and full reporting of outcomes
☒	☐	Estimates of effect sizes (e.g. Cohen's *d*, Pearson's *r*), indicating how they were calculated

Our web collection on statistics for biologists contains articles on many of the points above.

Software and code

Policy information about availability of computer code

Data collection	Nikon NIS Elements AR 5.41.02 for microscopy images.
Data analysis	GraphPad Prism Software, Version 9. Amersham ImageQuant 800 for southern blot signal quantification.

For manuscripts utilizing custom algorithms or software that are central to the research but not yet described in published literature, software must be made available to editors and reviewers. We strongly encourage code deposition in a community repository (e.g. GitHub). See the Nature Portfolio guidelines for submitting code & software for further information.

Data

Policy information about availability of data

All manuscripts must include a data availability statement. This statement should provide the following information, where applicable:

- Accession codes, unique identifiers, or web links for publicly available datasets
- A description of any restrictions on data availability
- For clinical datasets or third party data, please ensure that the statement adheres to our policy

All data generated or analyzed during this study as well as the uncropped western blots, are included in this article and its supplementary information files.

Research involving human participants, their data, or biological material

Policy information about studies with human participants or human data. See also policy information about sex, gender (identity/presentation), and sexual orientation and race, ethnicity and racism.

Reporting on sex and gender	N/A
Reporting on race, ethnicity, or other socially relevant groupings	N/A
Population characteristics	N/A
Recruitment	N/A
Ethics oversight	N/A

Note that full information on the approval of the study protocol must also be provided in the manuscript.

Field-specific reporting

Please select the one below that is the best fit for your research. If you are not sure, read the appropriate sections before making your selection.

☒ Life sciences ☐ Behavioural & social sciences ☐ Ecological, evolutionary & environmental sciences

For a reference copy of the document with all sections, see nature.com/documents/nr-reporting-summary-flat.pdf

Life sciences study design

All studies must disclose on these points even when the disclosure is negative.

Sample size	As is standard in the field, 200 to 300 nuclei were counted per conditions in all Immunofluorescence experiments .<br>As is standard in the field, 30 to 40 metaphases were analyzed per conditions in all metaphase spreads FISH analyses. Each experiments were performed at least 3 times independently. These informations are specified in the figure legends.
Data exclusions	No data were excluded from any analysis.
Replication	Each experiments were replicated at least 3 times with successful reproducibility as specified in the figure legends.
Randomization	Samples were allocated into the group depending on cell treatments as described in the manuscript. Due to relatively small group numbers, randomization was not employed during these experiments.
Blinding	Where possible (e.g. Metaphase analyses and immunofluorescence) samples were scored blindly. After slide mounting, a lab member not involved in the study would attribute numbers to each sample and disclose after analysis performed by the first author of the study.<br>For other experiment (e.g. southern blot, wester blot)blinding was not possible because samples were loaded on gels in logical ways that require the experimentalist to know the treatment conditions.

Reporting for specific materials, systems and methods

We require information from authors about some types of materials, experimental systems and methods used in many studies. Here, indicate whether each material, system or method listed is relevant to your study. If you are not sure if a list item applies to your research, read the appropriate section before selecting a response.

Materials & experimental systems

n/a	Involved in the study	
☐	☒	Antibodies
☐	☒	Eukaryotic cell lines
☒	☐	Palaeontology and archaeology
☒	☐	Animals and other organisms
☒	☐	Clinical data
☒	☐	Dual use research of concern
☒	☐	Plants

Methods

n/a	Involved in the study	
☒	☐	ChIP-seq
☒	☐	Flow cytometry
☒	☐	MRI-based neuroimaging

Antibodies

Antibodies used

Anti-Poly(ADP-Ribose) 10H mouse monoclonal Enzo Life Science Cat# ALX-802-220-R100; RRID:AB_2256326
Anti-PARP1 C-2-10 mouse monoclonal Enzo Life Science Cat#BML-SA249 BML-SA249/parp-1; RRID:AB_11001350
Anti-PARP-2 (4G8) mouse monoclonal Enzo Life Science Cat# ALX-804-639-L001
Anti-TRF2 rabbit polyclonal Novus biologicals Cat#NB110-57130 trf-2-antibody_nb110-57130; RRID:AB_844199
Anti-53BP1 (BP13) mouse monoclonal Millipore Cat# MAB3802
Anti-DYKDDDDK (FLAG) (FG4R) mouse monoclonal Invitrogen Cat# MA1-91878
Anti-phospho RPA32 (S4/S8) rabbit polyclonal Bethyl Cat# A300-254A-T
Anti-RAD51 rabbit monoclonal Abcam Cat# ab133534
Anti-POLD3 mouse monoclonal Abnova Cat# H00010714-M01
Anti-RFP rabbit polyclonal GeneTex Cat# GTX127897
Anti-DYKDDDDK Tag Antibody (FG4R) FLAG-tag mouse monoclonal ThermoFisher Cat# MAI-91878
Anti-BLM rabbit Gift from Dr. O'Sullivan lab (University of Pittsburgh) and Dr. Karlseder lab (Salk Institute).
Anti-beta Actin AC15 mouse monoclonal Sigma Cat# 088M4804
Secondary antibodies used are:
Abcam Goat Anti-Mouse IgG H+L (HRP) Cat#Ab6728
Abcam Goat anit-Rabbit IgG H+L (HRP) Cat#Ab6727
ThermoFisher Scientific Goat Anti-Mouse IgG H+L Highly Cross-Absorbed Secondary Antibodt Alexa Fluor TM Plus594 Cat#A32742
ThermoFisher Scientific Goat Anti-Rabbit IgG H+L Highly Cross-Absorbed Secondary Antibodt Alexa Fluor TM Plus594 Cat#32740

Validation

All antibodies, except for the anit-BLM antibody, are commercially available and validated by the manufacturers. The species are mentioned above and application can be accessed on the manufacturers' websites. These antibody validation have been confirmed on human lysates using western blot and immuno-fluorescence according to the product instructions. Below are the links:
Abcam: "Our Abpromise guarantee covers the use of ab133534 in the tested applications"
https://www.abcam.com/primary-antibodies/how-we-validate-our-antibodies

Enzo Life Sciences: "monoclonal and polyclonal antibodies [are] backed by peer-reviewed citations, our Worry-free Antibody Trial Program and expert technical support"
https://www.enzolifesciences.com/browse/products/by-product-type/antibodies/

Novus Biologicals: "Novus Biologicals Is a Trusted Leader in Quality Life Science Reagents. We Are Committed to Providing Researchers With the Highest Quality Antibodies" "To that end, we actively seek high quality, highly validated products and provide support to ensure that our customers have the tools to properly validate their own assays. We are also collaborating with several global initiatives that help life science researchers choose antibodies with proven results. Of the five pillars of validation established by these initiatives, genetic knockout validation provides the most reliable control for assessing antibody specificity"
https://www.novusbio.com/reproducibility.html

Millipore/Sigma: "MilliporeSigma's highly validated antibodies are guaranteed for quality performance. In addition to application-specific validation, all of our antibodies are backed with a best-in-industry technical service team dedicated to our antibodies customers.These efforts and collaborations have led to new validation techniques and novel antibody-based technologies, such as improved bead-based multiplex assays and imaging flow cytometry"
https://www.emdmillipore.com/US/en/life-science-research/antibodies-assays/antibodies-overview/Antibody-Development-and-Validation/cFOb.qB.8McAAAFOb64qQvSS,nav

Genetex: "GeneTex's version of the IWGAV plan follows the five standard strategies and includes (1) Knockout/Knockdown; (2) Comparable Antibodies; (3) Immunoprecipitation followed by Mass Spectrometry (IP/MS); (4) Biological and Orthogonal Validation; and (5) Recombinant Protein Expression"
https://www.genetex.com/Product/Overview/primary_antibodies

ThermoFisher: "To drive reagent quality, directly address antibody reproducibility, and help provide customers with confidence in our antibodies, Thermo Fisher Scientific has adopted three concepts to help improve experimental reproducibility and reporting based on the recommendations of the International Working Group for Antibody Validation (IWGAV):https://www.thermofisher.com/us/en/home/life-science/antibodies/invitrogen-antibody-validation.html"

In addition, antibodies used in PLA experiments have been tested in our lab individually in "single antibody" controls.
The BLM antibody was validated by our collaborators https://doi.org/10.1093/nar/gks407

Eukaryotic cell lines

Policy information about cell lines and Sex and Gender in Research

Cell line source(s)

All our cell lines are derived from the HeLaLT expressing telomere FAP developed in Dr. Opresko lab and is described previously: Fouquerel et al., Mol Cell 2019.

Authentication

Cell lines were authenticated previously (O'Sullivan et al., 2014; Fouquerel et la., 2019) . For derivatives of these cells generated in this study, the genotyping was verified by PCR amplification and sequencing of the relevant loci and protein levels established by western blotting with appropriate antibodies.

Mycoplasma contamination	All our cell Lines are tested for mycoplasma contamination on a monthly basis using LookOut kit to confirm that they test negative for mycoplasma infection. All cells lines used in this study tested negative for mycoplasma infection.
Commonly misidentified lines (See ICLAC register)	no commonly misidentified lines were used in this study.

Plants

Seed stocks	*Report on the source of all seed stocks or other plant material used. If applicable, state the seed stock centre and catalogue number. If plant specimens were collected from the field, describe the collection location, date and sampling procedures.*
Novel plant genotypes	*Describe the methods by which all novel plant genotypes were produced. This includes those generated by transgenic approaches, gene editing, chemical/radiation-based mutagenesis and hybridization. For transgenic lines, describe the transformation method, the number of independent lines analyzed and the generation upon which experiments were performed. For gene-edited lines, describe the editor used, the endogenous sequence targeted for editing, the targeting guide RNA sequence (if applicable) and how the editor was applied.*
Authentication	*Describe any authentication procedures for each seed stock used or novel genotype generated. Describe any experiments used to assess the effect of a mutation and, where applicable, how potential secondary effects (e.g. second site T-DNA insertions, mosiacism, off-target gene editing) were examined.*
